# *MtCLE08*, *MtCLE16,* and *MtCLE18* Transcription Patterns and Their Possible Functions in the Embryogenic Calli of *Medicago truncatula*

**DOI:** 10.3390/plants12030435

**Published:** 2023-01-17

**Authors:** Andrei A. Kudriashov, Natalia S. Zlydneva, Elena P. Efremova, Varvara E. Tvorogova, Ludmila A. Lutova

**Affiliations:** 1 Department of Genetics and Biotechnology, Saint Petersburg State University, 7/9 Universitetskaya Emb, 199034 Saint Petersburg, Russia; 2 Center for Genetic Technologies, N. I. Vavilov All-Russian Institute of Plant Genetic Resources (VIR), 42 Bolshaya Morskaya Street, 190000 Saint Petersburg, Russia; 3 Plant Biology and Biotechnology Department, Sirius University of Science and Technology, 1 Olympic Avenue, 354340 Sochi, Russia

**Keywords:** CLE peptides, plant regeneration, somatic embryogenesis, *Medicago truncatula*

## Abstract

CLE peptides are well-known hormonal regulators of plant development, but their role in somatic embryogenesis remains undetermined. *CLE* genes are often regulated by WOX transcription factors and, in their turn, regulate the expression level of *WOX* genes. In this study, we used in vitro cultivation, as well as qPCR and transcriptomic analysis, to find CLE peptides which could regulate the *MtWOX9-1* gene, stimulating somatic embryogenesis in *Medicago truncatula*. Three CLE peptides were found which could probably be such regulators, but none of them was found to influence *MtWOX9-1* expression in the embryogenic calli. Nevertheless, overexpression of one of *CLE* genes under study, *MtCLE16*, decreased somatic embryogenesis intensity. Additionally, overexpression of *MtCLE08* was found to suppress expression of *MtWOX13a*, a supposed antagonist of somatic embryo development. Our findings can be helpful for the search for new regeneration regulators which could be used for plant transformation.

## 1. Introduction

CLAVATA3/EMBRYO SURROUNDING REGION-RELATED (CLE) peptides are small peptide hormones regulating many aspects of plant development. The genes from the *CLE* family encode pre-propeptides, which, after proteolytic processing and post-translational modifications, turn into functional peptide hormones: CLE peptides. CLE peptides are 12–14 amino acids long and, as with other hormones, are used for intercellular communication [1]. Reception of CLE-peptides is carried out through leucine-rich repeat-receptor-like kinases from the CLAVATA1 (CLV1) and BARELY ANY MERISTEM (BAM) groups, but further regulatory pathways, by which CLE peptides may influence gene transcription, are unclear [2].

CLE peptides represent one of the most diverse groups of plant hormones. In *Medicago truncatula*, for example, 52 *CLE* genes were found [3]. Functions of CLE peptides are related to variable developmental processes, including meristem maintenance, embryogenesis, leaf development, nodulation, etc. [4]. The most well-known CLE peptide, CLAVATA3 (CLV3), restricts the proliferation of stem cells in the shoot apical meristem (SAM). The *CLV3* gene is expressed in the central zone of SAM, maintaining SAM size together with the WUSCHEL (WUS) transcription factor from the WUSCHEL-related homeobox (WOX) family. *WUS* is stably expressed in the organizing center. The protein it encodes migrates to the upper layers of the SAM and inhibits the differentiation of its cells, due to the activation and repression of different targets related to auxin and cytokinin signaling [5,6]. WUS also directly stimulates *CLV3* expression in the central zone of SAM, binding to the sequences located in a 3′-regulatory region of the *CLV3* gene [7]. CLV3, in its turn, represses *WUS* expression through LRR-kinases and similar proteins, including CLAVATA1 (CLV1) and RECEPTOR-LIKE PROTEIN KINASE 2 (RPK2). This repression makes it possible for SAM cells to begin differentiation [8,9].

Apart from the WUS-CLV3 regulatory loop, there are several other examples of *WOX* and *CLE* genes interacting. In the root apical meristem (RAM), WOX5 transcription factor inhibits the differentiation of columella stem cells. The CLE40 peptide acts antagonistically to WOX5, repressing the expression of quiescent center markers in the distal domain of RAM and activating *WOX5* expression in the proximal domain [10]. TDIF (TRACHEARY ELEMENT DIFFERENTIATION INHIBITORY FACTOR) CLE peptide, encoded by *CLE41* and *CLE44* genes, stimulates the expression of *WOX4* and *WOX14* genes in the lateral meristem, which is important for cell proliferation in this tissue [11].

Although there are a lot of studies elucidating the functions of CLE peptides in different plant organs, the role of these hormones in embryogenesis is poorly understood. Several *CLE* genes were shown to function in the embryo, including *CLV3* [12], *CLE25* [13] and others, but the only known CLE peptide with specific functions during embryogenesis is CLE8. The *CLE8* gene is expressed in the embryo and in the endosperm. In plants with a loss of *CLE8* function, the endosperm is underdeveloped, and the embryo and suspensor develop abnormally. CLE8 is required for the activation of the *WOX8* gene in the suspensor and endosperm; overexpression of *CLE8* leads to an increase in seed size, but loss of *WOX8* function neutralizes this effect, suggesting that these two genes act together during embryogenesis regulation [14].

Many embryogenesis regulators also function during the similar process of somatic embryogenesis. Somatic embryogenesis (SE) is a process during which plant somatic cells develop into embryos which eventually can form whole plants. Usually, SE is observed in vitro, although for some plants (for example, some Kalanchoe species) it is also characteristic in vivo. It has been established that in vitro SE can be induced mostly by phytohormones and stress conditions, although many varied specific factors may exert an impact on the intensity and rate of this process. Typical protocols for SE induction include the cultivation of explants on a medium, containing auxins and cytokinins. As a way of plant regeneration, SE is widely used for transformation and propagation of plants, therefore, the search for regulators of this process is an important task for biotechnology.

Recently, a WOX transcription factor MtWOX9-1 was shown to stimulate SE in *M. truncatula* [15]. In this study, we found 3 CLE peptides possibly related with MtWOX9-1 and investigated their functions.

## 2. Results

### 2.1. Search for CLE Peptides Related with MtWOX9-1

**Figure 1:**
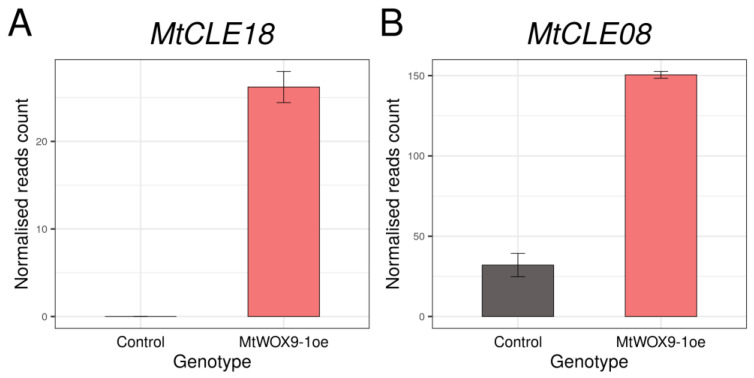
Expression levels of *MtCLE18* (**A**) and *MtCLE08* (**B**) in control (R108) calli and calli with *MtWOX9-1* overexpression according to the transcriptomic analysis [17]. Error bars represent the standard error for two biological replicates.

*MtWOX9-1* was found to be expressed during SE [16] and to stimulate this process [15]. Recently, transcriptomic analysis of calli with *MtWOX9-1* overexpression was performed [17]. We analyzed these data and found that *MtCLE18* gene expression level increases substantially in the calli with *MtWOX9-1* overexpression in comparison with the wildtype calli (Figure 1A, Table S1).

**Figure 2:**
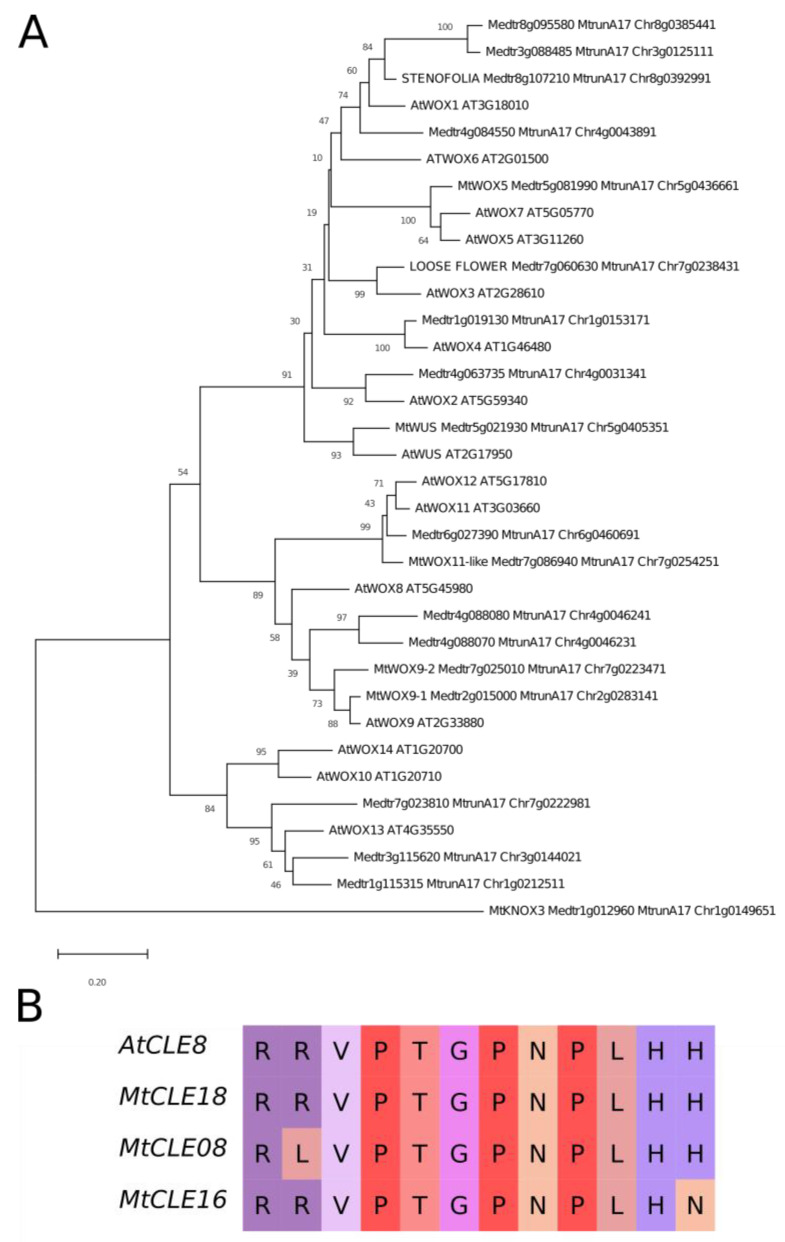
(**A**) Phylogenetic tree of *A. thaliana* and *M. truncatula* WOX proteins, based on homeodomain sequences. The tree was inferred using the Neighbor-Joining method [18]. The percentages of replicate trees in which the associated proteins are clustered together in the bootstrap test (500 replicates) are shown next to the branches [19]. The tree is drawn to scale, with branch lengths in the same units as those of the evolutionary distances used to infer the phylogenetic tree. Evolutionary analyses were conducted in MEGA X [20]; (**B**) Alignment of CLE domains of *A. thaliana* CLE8 peptide and *M. truncatula* MtCLE18, 08 and 16 peptides. Different colors represent different amino acids.

It was found by Fiume and Fletcher [14] that, during zygotic embryogenesis in *A. thaliana*, the CLE8 peptide stimulates expression of the *WOX8* gene. Interestingly, the *MtWOX9-1* gene is closely related with *WOX8* and *WOX9* genes of *A. thaliana* (Figure 2A), whereas MtCLE18 has a CLE domain identical to the CLE domain of the CLE8 peptide (Figure 2B). Therefore, we supposed that *MtCLE18*, whose expression level is affected by MtWOX9-1, may also have influence on *MtWOX9-1* expression, and, thereby, on SE intensity. We also found that, among MtCLE peptides, two other CLE exist—MtCLE08 and MtCLE16—with CLE domains similar to MtCLE18: their CLE domains differ from MtCLE18′s in one amino acid only (Figure 2B). Moreover, *MtWOX9-1* overexpression also increases the expression level of *MtCLE08* according to the transcriptomic analysis (Figure 1B, Table S1). Thus, in this study, we tried to check the expression patterns of *MtCLE08*, *16,* and *18*, as well as to evaluate the influence of their overexpression on SE capacity and *MtWOX9-1* expression.

### 2.2. MtCLE08, 16 and 18 Expression Patterns in Different Organs

**Figure 3:**
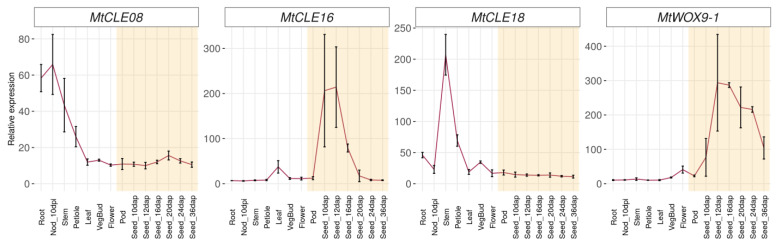
Expression patterns of *MtCLE08*, *MtCLE16*, *MtCLE18* and *MtWOX9-1* genes in different organs and during different stages of seed development according to the Medicago truncatula Gene Expression Atlas [21]. The error bars represent SD of three biological replicates. Stages of seed development are highlighted in yellow.

To investigate the possible functions of *MtCLE08*, *16,* and *18*, we evaluated their expression patterns, as well as the expression pattern of *MtWOX9-1* gene, in different organs using the Medicago truncatula Gene Expression Atlas [21] (Figure 3). According to this database, *MtWOX9-1* is mostly expressed in generative organs, seeds, pods and flowers, which is consistent with its high expression level in ovules as evaluated with qPCR [16]. Among the *MtCLE* genes investigated, only *MtCLE16* has a high expression level in seeds, whereas *MtCLE18* is active in the stem, roots and vegetative buds, and *MtCLE08* is expressed in nodules, roots, stem, and petioles, with low expression levels in other organs. Therefore, according to the expression patterns in vivo, MtCLE16 is more likely to be involved in the embryogenesis process than MtCLE08 and 18.

### 2.3. Evaluation of the Expression Dynamics of MtCLE08, 16, and 18 during Somatic Embryogenesis

We also evaluated the expression of the *MtCLE* genes under study in embryogenic and non-embryogenic calli. For that purpose, we used calli of the embryogenic 2HA *M. truncatula* line and its predecessor, non-embryogenic A17 line (Figure S1A,B). We measured the expression of the *MtCLE* genes in explants and calli of both lines, which were cultured as described in Section 4, in the conditions, inducing SE in the 2HA line. As a result, only *MtCLE08* and *18* were expressed in these in vitro conditions.

**Figure 4:**
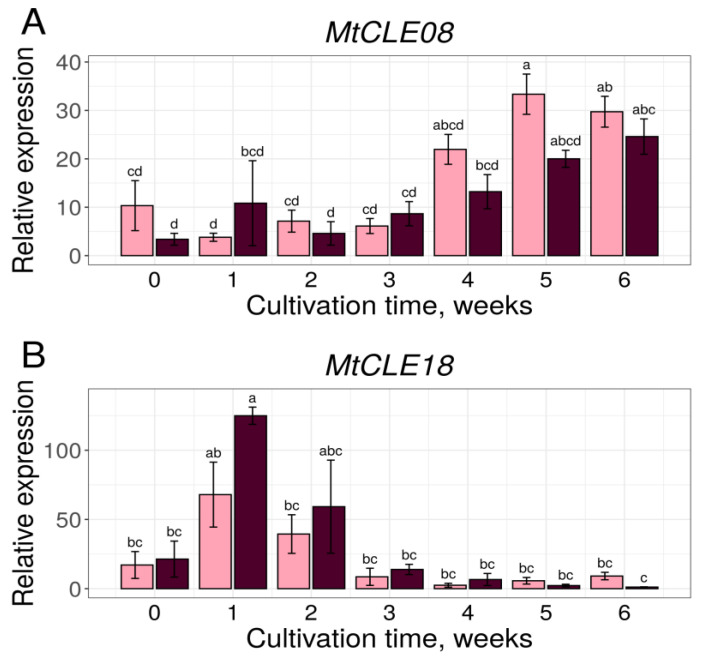
*MtCLE08* (**A**) and *MtCLE18* (**B**) genes expression dynamics during in vitro cultivation of explants of embryogenic 2HA (pink) and non-embryogenic A17 (dark-red) lines. Error bars represent the standard error. Data are obtained from three biological replicates. To assess the statistical significance of the observed differences, one-way analysis of variance (one-way ANOVA) with Tukey’s post hoc test was used, with confidence level 0.95. Different lowercase letters represent expression levels with statistically significant differences (*p*-value < 0.05).

The *MtCLE08* gene expression increased during the later stages of cultivation (4–6th week), when somatic embryos developed on the calli of the embryogenic 2HA line. The expression increase was found in both the embryogenic and non-embryogenic lines at these stages, although it occurred earlier and more prominently in the 2HA line (Figure 4A). At the same time, *MtCLE18* was expressed predominantly at the early stages of callus development in both lines (Figure 4B). We did not observe the expression of *MtCLE16* in calli of the A17 and 2HA lines (data not shown), which is consistent with transcriptome analysis results, showing an almost zero expression level of *MtCLE16* in the calli of another embryogenic *M. truncatula* line, R108 (Table S1).

### 2.4. Evaluation of the Influence of MtCLE08, 16, and 18 Overexpression on MtWOX Expression Levels

**Figure 5:**
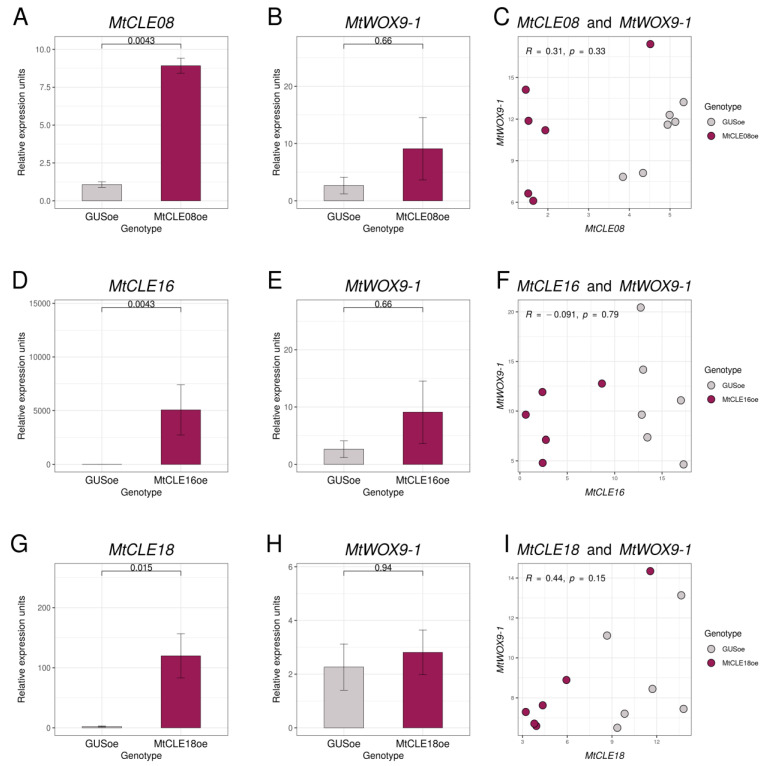
Expression levels of *MtCLE08* (**A**), *MtCLE16* (**D**), *MtCLE18* (**G**), and *MtWOX9-1* (**B**,**E**,**H**) genes in transgenic calli with *GUS* and *MtCLE08* (**A**,**B**), *MtCLE16* (**D**,**E**), or *MtCLE18* (**F**,**G**) overexpression. Expression levels of the *MtCLE08* (**C**), *MtCLE16* (**F**), or *MtCLE18* (**I**) gene and the *MtWOX9-1* gene in individual callus samples. Error bars represent the standard error. Data are obtained from six biological repeats per genotype. To assess the statistical significance of the observed differences between different genotypes of calli, the Wilcoxon signed-rank test was used. To assess the statistical significance of correlation between expression levels of different genes, Spearman’s rank correlation test was used. Material for gene expression analysis in T0 calli was taken at 43rd–56th day after transformation, after 33–50 days of cultivation on the callus induction medium, and 7–14 days of cultivation on the hormone-free medium.

To check if chosen CLE peptides have any influence on *MtWOX9-1* expression, we transformed leaf explants of the *M. truncatula* R108 line with constructions for *MtCLE08*, *16,* or *18* overexpression, as well as the construction for *GUS* overexpression, which was used as a control. After 30–50 days of cultivation on the selective medium with hormones (BAP and 2.4-D), transgenic calli developed, and we transferred them onto the hormone-free selective medium. After several days of cultivation on the hormone-free medium, we extracted the material for expression analysis. According to our qPCR analysis, T0 calli had elevated expression levels of corresponding *CLE* genes, but none of these genes was found to have any influence on the *MtWOX9-1* expression level (Figure 5A,B,D,E,G,H). Due to the heterogeneous nature of transgenic T0 calli, individual calli transformed with construction for some *CLE* gene overexpression may have different levels of expression for this *CLE* gene. Therefore, we also evaluated the correlation between *MtWOX9-1* and the analyzed *CLE* gene expression in transgenic calli, but no significant correlation was found (Figure 5C,F,I).

**Figure 6:**
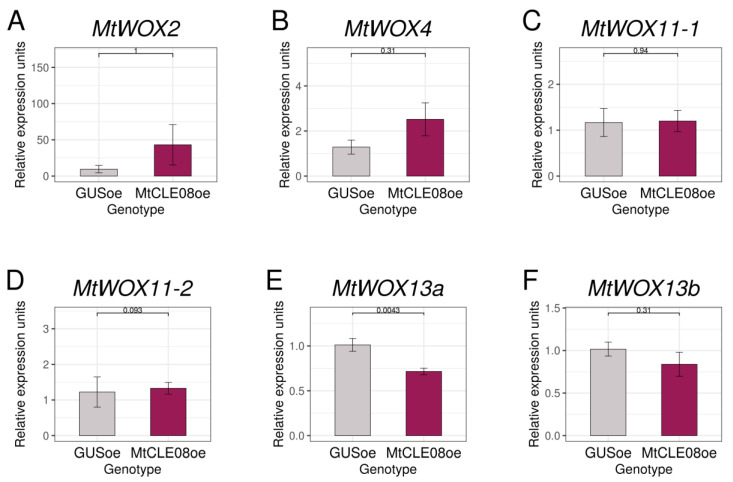
Expression levels of the *MtWOX2* (**A**), *MtWOX4* (**B**), *MtWOX11-1* (**C**), *MtWOX11-2* (**D**)*, MtWOX13a* (**E**) and *MtWOX13b* (**F**) genes in transgenic calli with *GUS* and *MtCLE08* overexpression. Error bars represent the standard error. Data are obtained from 6 biological repeats per genotype. To assess the statistical significance of the observed differences between different genotypes of calli, the Wilcoxon signed-rank test was used. Material for gene expression analysis in T0 calli was taken at 43rd–56th day after transformation, after 33–50 days of cultivation on the callus induction medium and 7–14 days of cultivation on the hormone-free medium.

We also suggested that MtCLE08, 16, or 18 could change expression of other *WOX* genes, on which *MtWOX9-1* overexpression has influence. According to the transcriptomic analysis, *WOX* genes which changed expression in *MtWOX9-1*-overexpressing calli (*p*-value < 0.05) included *MtWOX2, MtWOX4*, *MtWOX11-1,* and *MtWOX13a* (Table S1). We also analyzed *MtWOX11-2* and *MtWOX13b*, which had rather high expression levels in both R108 (wildtype) and *MtWOX9-1*-overexpressing calli (DESeq2 normalized reads mean >50). Using qPCR, we checked the expression levels of these *WOX* genes in control calli and calli with *MtCLE08*, *16,* or *18* overexpression. We did not find any significant influence in most cases, but *MtWOX13a* gene expression level was lower in the calli with *MtCLE08* overexpression in comparison with control *GUS* overexpressing calli (*p*-value = 0.0043, Wilcoxon test) (Figure 6, Figures S2 and S3).

**Figure 7:**
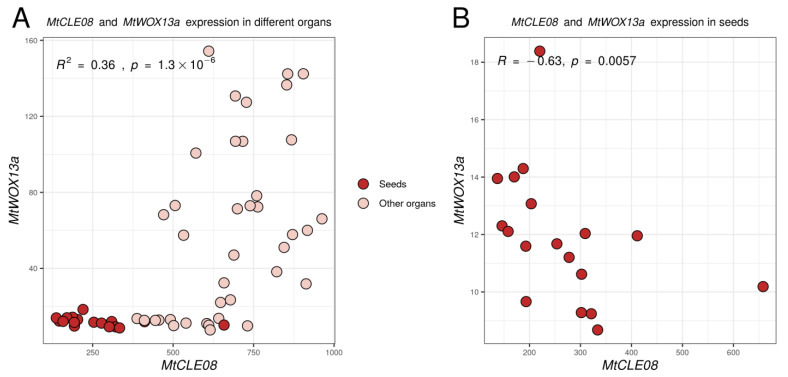
Expression levels of the *MtWOX13a* and *MtCLE08* genes in different organs and during different stages of seed development according to the Medicago truncatula Gene Expression Atlas [21]. To assess the statistical significance of correlation between expression levels of different genes, Spearman’s rank correlation test was used.

Given that in this experiment 18 different expression comparisons were made, as the effect of three *CLE* genes was evaluated for six *WOX* genes, the Bonferroni adjusted *p*-value for the difference between *MtWOX13a* expression in control and MtCLE08oe calli is 0.0043 × 18 = 0.0774, which exceeds the traditionally accepted threshold level (0.05). To check if the relation between *MtCLE08* and *MtWOX13a* genes exists, we evaluated their expression in different *M. truncatula* organs using the Medicago truncatula Expression Atlas [21]. Interestingly, expression of these two genes had a significant positive correlation when all organs were analyzed (R = 0.6, *p*-value = 1.3 × 10^−0.6^), but also a significant negative correlation in seeds only (R = −0.63, *p*-value = 0.0057) (Figure 7), suggesting the existence of complex regulatory intercourse between these two genes.

### 2.5. Evaluation of the Influence of MtCLE08, 16, and 18 Overexpression on Somatic Embryogenesis

**Figure 8:**
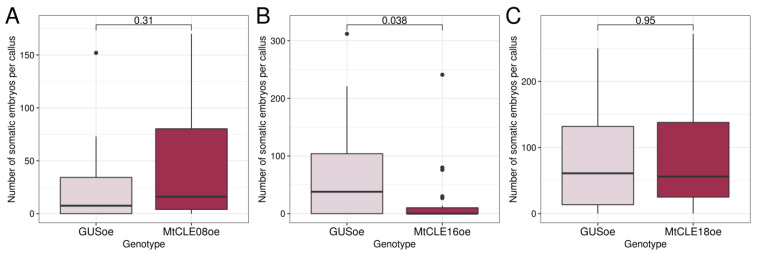
Number of somatic embryos per callus in transgenic calli with *GUS* and *MtCLE08* (**A**), *MtCLE16* (**B**)*,* or *MtCLE18* (**C**) overexpression. Data are obtained from 10–24 calli for different samples. For *MtCLE16* overexpressing calli, two experiments were performed with similar results. To assess the statistical significance of the observed differences, the Wilcoxon signed-rank test was used. Analysis of *M. truncatula* SE capacity and weight measurement of T0 calli were performed on the 72nd–86th day after transformation, after 33–50 days of cultivation on the callus induction medium and 30–43 days of cultivation on the hormone-free medium.

Although the overexpression of *MtCLE08*, *16,* or *18* appeared not to have any influence on the *MtWOX9-1* expression level, we checked whether overexpression of these genes had any effect on the SE and callus formation capacity of T0 calli (Figure S1C,D). No influence of the overexpression of any gene analyzed on callus weight was found (data not shown). We also did not detect any effect of *MtCLE08* or *MtCLE18* overexpression on embryogenic calli development, but *MtCLE16* overexpression significantly reduced the number of somatic embryos per callus (Figure 8).

## 3. Discussion

Transcription factors from the WOX family regulate cell proliferation and differentiation, and different *WOX* genes have been found to stimulate regeneration processes. For example, WUS or its orthologs have been shown to induce regeneration in many plant species and can be applied as morphogenic regulators to speed up the transformation process or to make it possible [22,23]. WOX2 and 8 induce regeneration in tobacco [24], and MtWOX9-1 has been shown to stimulate SE in *M. truncatula* [15]. In this study, using *M. truncatula*, we tried to find new participants of SE among the family of CLE peptides, well-known regulators of the expression of the *WOX* genes. As *WOX* and *CLE* genes can be related to each other through feedback regulatory loops, we supposed that *CLE* genes regulated by MtWOX9-1 may possibly, in their turn, have an influence on *MtWOX9-1* expression level. Using transcriptomic data, we found that *MtWOX9-1* overexpression led to the increase in expression levels in *MtCLE18* and *MtCLE08* genes. MtCLE18 and MtCLE08 CLE domains, as well as the CLE domain of another peptide, MtCLE16, are similar to the CLE domain of the *A. thaliana* CLE8 peptide, for which functions in embryogenesis have been shown, as well as its stimulatory effect on the expression of *WOX8*, a close homolog of *MtWOX9-1* [14]. We supposed that MtCLE08, MtCLE16 and/or MtCLE18 may participate in zygotic or somatic embryogenesis and influence *MtWOX9-1* expression. To check our hypotheses, we evaluated the expression levels of chosen CLE peptides in different plant organs, as well as in callus cultures. Interestingly, the *MtCLE16* gene, having specific expression increase in developing seeds, did not demonstrate any significant expression during somatic embryogenesis. Localization of its expression in seeds as well as mutant analysis may help to elucidate the functions of this gene. At the same time, *MtCLE18* was specifically expressed in the stem as well as at the early stages of callus development in both embryogenic and non-embryogenic lines. Possibly, its functions may be related to the activity of lateral meristems, which play a significant part in callus development [25]. In its turn, *MtCLE08* demonstrated a less specific expression pattern, being activated in different plant organs including roots, nodules, and the stem, as well as in the calli of both embryogenic and non-embryogenic lines at different stages. Nevertheless, the increase in its expression level during SE suggests its participation in this process.

We did not find any influence of overexpression of the *CLE* genes under study on the *MtWOX9-1* expression level. This result suggests that the relations between CLE8 and WOX8 homologs are not very conservative. It is also worth mentioning that there are several *WOX* genes from the *WOX8/9* group in *M. truncatula*, and all of them, including *MtWOX9-1*, are more closely related to *WOX9* than to *WOX8* (Figure 2). It would be interesting to check if MtCLE08, 16, or 18 peptides have any influence on the expression of other *WOX* genes from this group, although these *WOX* genes are not expressed in wild type embryogenic calli (Table S1). 

We also suggest that the effect of *MtCLE08* and *18* overexpression could have been missed because their expression level was rather high even in control calli. It is also possible that these peptides can stimulate *MtWOX9-1* expression in different conditions. Probably, it is worth investigating whether the induction of *MtCLE08* and *18* in the tissues where they are normally not expressed has any effect on *MtWOX9-1* expression or other processes.

The effect of *MtCLE08* overexpression on *MtWOX13a* expression level was found. MtWOX13a is a member of an ancient WOX family branch. Its ortholog in *A. thaliana* is expressed in many tissues and was studied primarily as the regulator of fruit development [26]. Its functions in *M. truncatula* have yet to be discovered, but the expression level of *MtWOX13a* is lower in *MtWOX9-1*-overexpressing highly embryogenic calli than in control calli (Table S1). This may suggest that MtWOX13a acts antagonistically to SE.

We did not find a SE stimulator among MtCLE08, 16 and 18; on the contrary, *MtCLE16* overexpression repressed SE. A recent study shows that several CLE peptides (CLE1-7) repress shoot regeneration in *A. thaliana* through the inhibition of *WUS* expression [27], but the sequences of their CLE domains differ significantly from the CLE domain of MtCLE16. In another study, *MtCLV3* expression was found to be associated with SE in *M. truncatula* [28], but, so far, no regeneration stimulator has been found among CLE peptides. 

The mechanisms by which *MtCLE16* overexpression represses embryogenesis are yet to be discovered, but one can suppose that MtCLE16 acts antagonistically to another, as yet unknown, CLE peptide, which stimulates regeneration. If such a peptide was found, it could probably be used as a morphogenic regulator to enhance transformation efficiency. The MtCLE16 CLE domain has an asparagine residue in the 12th position, whereas the MtCLE08 and 18 CLE domains have a histidine residue. The *MtCLE16* overexpression effect and the absence of significant effects of *MtCLE08* and *18* overexpression allow us to suggest that this amino acid position is important in SE regulation by CLE peptides. It would be interesting to investigate whether overexpression of a mutated *MtCLE18* gene encoding peptide with asparagine residue at the 12th position could have repressive effect on SE. 

Taken together, our results specify regulatory relationships between *WOX* and *CLE* genes in the embryogenic calli and suppose several directions for the search of new morphogenic regulators.

## 4. Materials and Methods

### 4.1. Plants and Microorganisms

*M. truncatula* R108, A17 and 2HA lines were used. Seeds of the R108 line were provided by the Samuel Roberts Institute (OK, USA). Seeds of the 2HA line were provided by Dr. Mireille Chabaud (Laboratory of Plant-Microbe Interactions, Ausville-Tolozan, France). Seeds of the A17 line were provided by colleagues from Wageningen University (Netherlands). 

Prior to germination, *M. truncatula* seeds were submerged in sulfuric acid (95–97%) for 10 min, rinsed 10 times with distilled water, and put on the 1% agar at 4 °C. Plants were grown in in vivo conditions in soil (Terra Vita, Nord Pulp, Russia) mixed with vermiculite (3:1).

Transformation of R108 plants, induction of SE in the 2HA line, and in vitro cultivation of the A17 line were carried out as in [15]. Evaluation of SE capacity was performed in the R108 line T0 calli, which were developed after leaf explant transformation, without obtaining transgenic plants. Embryo number per callus and callus weight were evaluated for 10–24 T0 calli per genotype, depending on the experiment. Analysis of *M. truncatula* SE capacity and weight measurement for T0 calli were performed on the 72nd–86th day after transformation, after 33–50 days of cultivation on the callus induction medium and 30–43 days of cultivation on the hormone-free medium. Material for gene expression analysis in T0 calli was taken on the 43rd–56th day after transformation, after 33–50 days of cultivation on the callus induction medium and 7–14 days of cultivation on the hormone-free medium. 

Escherichia coli DH10B strain was used for cloning. For *M. truncatula* transformation, *Rhizobium radiobacter* (*Agrobacterium tumefaciens*) AGL1 strain was used. Microorganisms cultivation conditions and transformation methods are described in [29].

### 4.2. Molecular Cloning and qPCR Analysis

To obtain the constructions for *MtCLE* genes overexpression, the CDS of *MtCLE* genes under study were cloned in the pDONR207 vector (Invitrogen) and then to the pMDC32 vector [30], using the Gateway method (Invitrogen).

For the expression analysis, RNA was isolated from plant calli using RNeasy Plant Mini Kit (Qiagen, Hilden, Germany) or TRIzol (Thermo Fisher Scientific, MA, USA), according to the manufacturer’s instructions. For DNAse treatment and DNAse removal, RapidOut DNA Removal Kit (Thermo Fisher Scientific) was used. Depending on the experiment, 50–500 ng of RNA was used for cDNA synthesis. For reverse transcription, RevertAid reverse transcriptase, RiboLock RNase Inhibitor (Thermo Fisher Scientific), and oligo-dT18 primer were used according to manufacturer’s instructions. Reverse transcription was performed in 20 μL volume; cDNA samples were diluted 5-fold with water. For each qPCR reaction, we used 1/100th of cDNA, synthesized in each reverse transcription reaction.

The kit with Eva Green (Syntol, Moscow, Russia) was used to perform qPCR, according to the manufacturer’s instructions. qPCR was performed in the CFX96 Real-Time PCR Detection System (Bio-Rad, CA, USA). The CFX-Manager software (Bio-Rad) was used for threshold cycle estimation. Quantitative analysis of differences in gene expression between samples was performed using the delta delta Ct method [31]. For gene expression analysis in T0 calli, 6 biological replicates (6 separate callus samples) per genotype were analyzed with qPCR. For the evaluation of gene expression dynamics in 2HA and A17 calli, 3 biological replicates (3 separate callus samples) were analyzed per each cultivation stage for each line.

For correlation analysis, delta Ct values of two analyzed genes were estimated for each sample, and then Spearman’s rank correlation coefficient was calculated. The *MtH3L* gene was used as a reference. Primers (Evrogen, Moscow, Russia) used in the study are shown in Table S2.

### 4.3. Software

For sequence analysis, Ugene [32], SnapGene (from GSL Biotech; available at snapgene.com) and ApE (M. Wayne Davis) were used. For statistical analysis and diagram drawing, Rstudio (RStudio Team, 2020) was used. MEGA X software [20] was used for alignment and phylogeny analysis.

## Data Availability

Raw data files and gene expression data on the transcriptome analysis of wild type calli and calli with *MtWOX9-1* overexpression are available via the Gene Expression Omnibus (GEO) with identifier GSE201314.

## Supplementary Materials

The following supporting information can be downloaded at: https://www.mdpi.com/article/10.3390/plants12030435/s1, Table S1: Results of transcriptomic analysis of calli with *MtWOX9-1* overexpression for chosen *MtCLE* and *MtWOX* genes. *WOX* genes chosen for analysis are highlighted with red. Table S2: Primers used in the study. Gene IDs in tables correspond to the versions MedtrA17_4.0 [33] and MtrunA17r5.0-ANR [34,35] of *M. truncatula* genome. Figure S1: (A,B) Representative photos of A17 (A) and 2HA (B) calli at the sixth week of cultivation. Somatic embryos on 2HA calli appear as green-colored structures. (C,D) Representative photos of T0 control calli with *GUS* overexpression, taken when the weighing and counting of embryos was performed (83th day of cultivation). Figure S2: Expression levels of the *MtWOX2* (A), *MtWOX4* (B), *MtWOX11-1* (C), *MtWOX11-2* (D), *MtWOX13a* (E) and *MtWOX13b* (F) genes in transgenic calli with *GUS* and *MtCLE16* overexpression. Error bars represent the standard error. Data are obtained from 6 biological repeats per genotype. To assess the statistical significance of the observed differences between different genotypes of calli, the Wilcoxon signed-rank test was used. Figure S3: Expression levels of the *MtWOX2* (A), *MtWOX4* (B), *MtWOX11-1* (C), *MtWOX11-2* (D), *MtWOX13a* (E) and *MtWOX13b* (F) genes in transgenic calli with *GUS* and *MtCLE18* overexpression. Error bars represent the standard error. Data are obtained from 4–6 biological repeats per genotype. To assess the statistical significance of the observed differences between different genotypes of calli, the Wilcoxon signed-rank test was used.
