# Supplementary figures and images for: MtCLE08, MtCLE16, and MtCLE18 Transcription Patterns and Their Possible Functions in the Embryogenic Calli of Medicago truncatula
Source: Plants (Basel). 2023 Jan 17;12(3):435. doi: 10.3390/plants12030435 (PMC9921462; doi:10.3390/plants12030435)

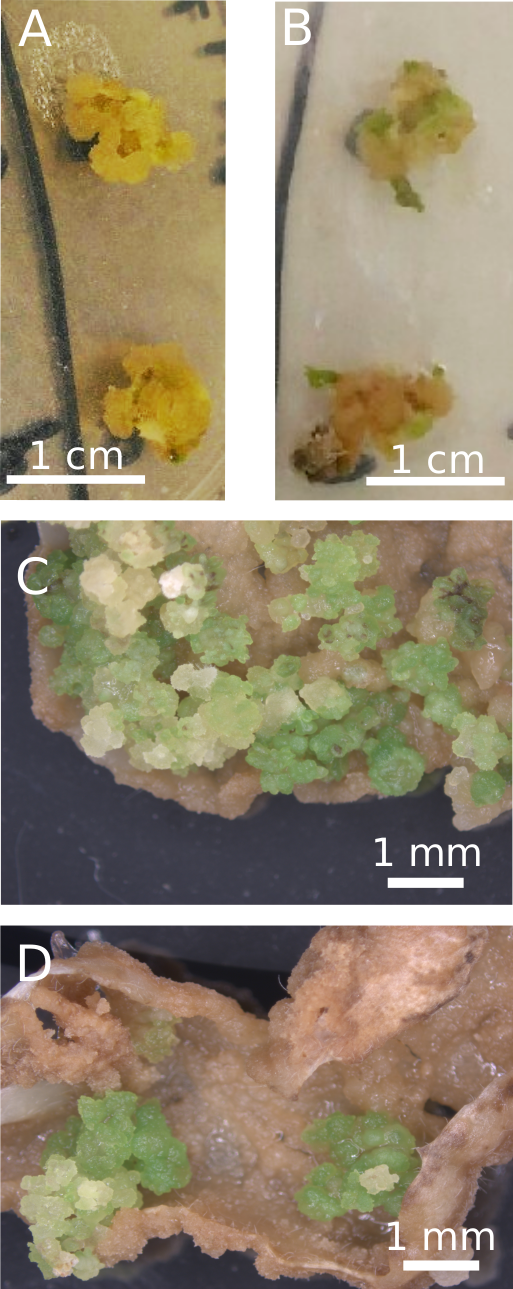

Supplement: Supplementary file 1 [file plants-12-00435-s001.zip › FigS1.tif]
